# Innovations in Public Financing for Family Planning at Subnational Levels: Sustainable Cofinancing Strategies for Family Planning With Nigerian States

**DOI:** 10.9745/GHSP-D-22-00242

**Published:** 2024-05-21

**Authors:** Victor Igharo, Uduak Ananaba, Olukunle Omotoso, TrishAnn Davis, Mwikali Kioko, Clea Finkle

**Affiliations:** a The Challenge Initiative, Nigeria Hub, Johns Hopkins Center for Communication Programs, Abuja, Nigeria.; b Johns Hopkins Center for Communication Programs, Baltimore, MD, USA.; c Independent consultant, Seattle, WA, USA.

## Abstract

Global declines in donor funding present a substantial threat to development financing in low- and middle-income countries. In Nigeria, the resources required to achieve states’ health goals surpass existing government budgets and available donor funding, a shortfall that incentivizes efforts to expand nondonor sources of financing, including public-driven cofinancing models. The Challenge Initiative (TCI) in Nigeria implements a demand-led model wherein 13 state governments requested technical support from TCI to adapt and scale up high-impact family planning and reproductive health (FP/RH) interventions. TCI provides a blend of technical coaching and financial support through the Challenge Fund, a mechanism designed to incentivize domestic funding for FP programming. To qualify as a recipient, states must demonstrate political will, financial commitment, and potential for impact at scale. However, state financial commitments alone are insufficient to guarantee the successful implementation of health scale-up initiatives. For this reason, the TCI Nigeria cofinancing strategy builds positive relations among key actors (donors, implementers, and government) and improves accountability in FP/RH financing. Although there are several donor-led cofinancing primary health care initiatives in Nigeria, such as the Saving One Million Lives Performance for Results project and Basic Healthcare Provision Fund, little is known about the role of government in driving the process specifically for improving domestic FP/RH financing. In Nigeria, state governments, in collaboration with TCI, developed a cofinancing model that helps states meet their FP/RH financing commitments. To promote effectiveness and sustainability, this model operates within an existing state structure, the State Annual Operation Plan. TCI’s cofinancing model motivates continuous improvement in state governments’ fiscal capacity, using a framework to measure, track, and reward financial and nonfinancial state commitments. Although the model is not a replacement for existing program tracking and monitoring tools, it helps subnational governments better harness their resources to accelerate improvement in FP/RH outcomes.

## Abstract (precis)

Using a cofinancing strategy, we present an innovative model on how local governments can collaborate with partners to harness all available resources and improve accountability through transparent agreement and documentation for sustainable development programming.

## Teaser

**Key Findings:**

- From 2018 to 2022, The Challenge Initiative (TCI) employed a cofinancing strategy in Nigeria to support states in their family planning and reproductive health (FP/RH) budget allocations and expenditures.
- Thirteen states accessed and used a total of 2.03 billion Nigerian naira (US$5.6 million) from the TCI Challenge Fund, a mechanism to incentivize domestic funding for FP programming.
- State governments demonstrated substantial improvements in their fiscal commitment and releases with a 4-year average of 78.7% release of committed resources toward FP programs.

**Key Implications:**

- The TCI Nigeria cofinancing model is a viable tool for building relations among key actors and improving accountability in RH financing.
- At the health systems level, this model can guide governments to allocate FP budgets within strategic planning frameworks for more sustainable funding.
- As a mechanism for mobilizing domestic resources, strengthening accountability systems, and building consensus and transparency, the model should be tested in other health and development areas beyond FP/RH.

## INTRODUCTION

Global declines in donor funding present a substantial threat to development financing in low- and middle-income countries.^1^ This is no less true in Nigeria, which faces inadequate domestic financing for health. Over the past decade, the federal and state governments of Nigeria have budgeted significant resources to develop and implement strategies to create an effective health care delivery system, but limited financing perennially stymies these goals. In 2001, African Union countries pledged to set a target of allocating at least 15% of their annual budget to improve the health sector.^2^ Nigeria’s expenditure on health as a share of gross domestic product and of general government expenditure is currently 3% and 3.8%, respectively.^3^ Although overall health spending has modestly increased from 1.19 billion Nigerian naira (N) (US$2.7 million) in 2019 to N1.477 billion (US$3.3 million) in 2021, the proportion of actual spending compared to current health budget stood at 15.9% in 2019.^4^ Inadequate funding is aggravated by weak governance, accountability, and policy implementation combined with limited data available for planning and decision-making. For these reasons, Nigeria’s health system ranked 187 of 195 countries in the 2018 health access quality index.^5^

Funding challenges are compounded because the benefits of investment in family planning (FP) for health and development outcomes are often poorly understood by high-level actors even though it is cross cutting in the health and social development pillars within the National Strategic Development Plan.^6^ Even when the government makes public funds for FP available through its fiscal commitments and policy, releases are often suboptimal and late, leading to significant implementation delay, low availability of consumables, deferred distribution of commodities to the last mile, poor demand, and overall weak access to quality reproductive health (RH) services.^7^ Together, these challenges have constrained improvements in health outcomes, especially the core Sustainable Development Goal (SDG-3) targets, and reduced motivation to advocate for and invest in FP.^8^

New approaches are needed to mobilize and routinize domestic funding flows while leveraging limited donor funds in catalytic ways. Governments and donors must collaborate to ensure that limited resources are strategically aligned to the highest impact approaches and focused on the lowest income populations to optimize investments.^9^ Recent funding deficits lend a sense of urgency to expanding nondonor sources of financing, especially through public financing and leveraged cofinancing models.^10^

New approaches are needed to mobilize and routinize domestic funding flows while leveraging limited donor funds in catalytic ways.

A cofinancing strategy can help local governments build and nurture collaborative relationships with partners to mobilize all available resources and improve accountability through transparent agreements and documentation for sustainable programming. Though health investment cofinancing has been around for decades,^11^^–^^13^ most governments in low-income countries have not fully taken advantage of the opportunities it presents. An effective cofinancing strategy helps to sustain health investments and gains made by state governments, facilitate fiscal accountability, and reduce dependence on donor funds.

Donors and governments in Nigeria increasingly recognize that local leadership should drive domestic financing as a key to sustaining gains of FP/RH.^14^ In Nigeria, The Challenge Initiative (TCI) supports states to take advantage of the benefits of cofinancing to mobilize domestic financing for FP programs. We describe TCI’s cofinancing model and innovations in Nigeria and present key results, learnings, and recommendations based on the implementation of the model in varying local government contexts.

## TCI PROGRAM IN NIGERIA

**FIGURE 1:**
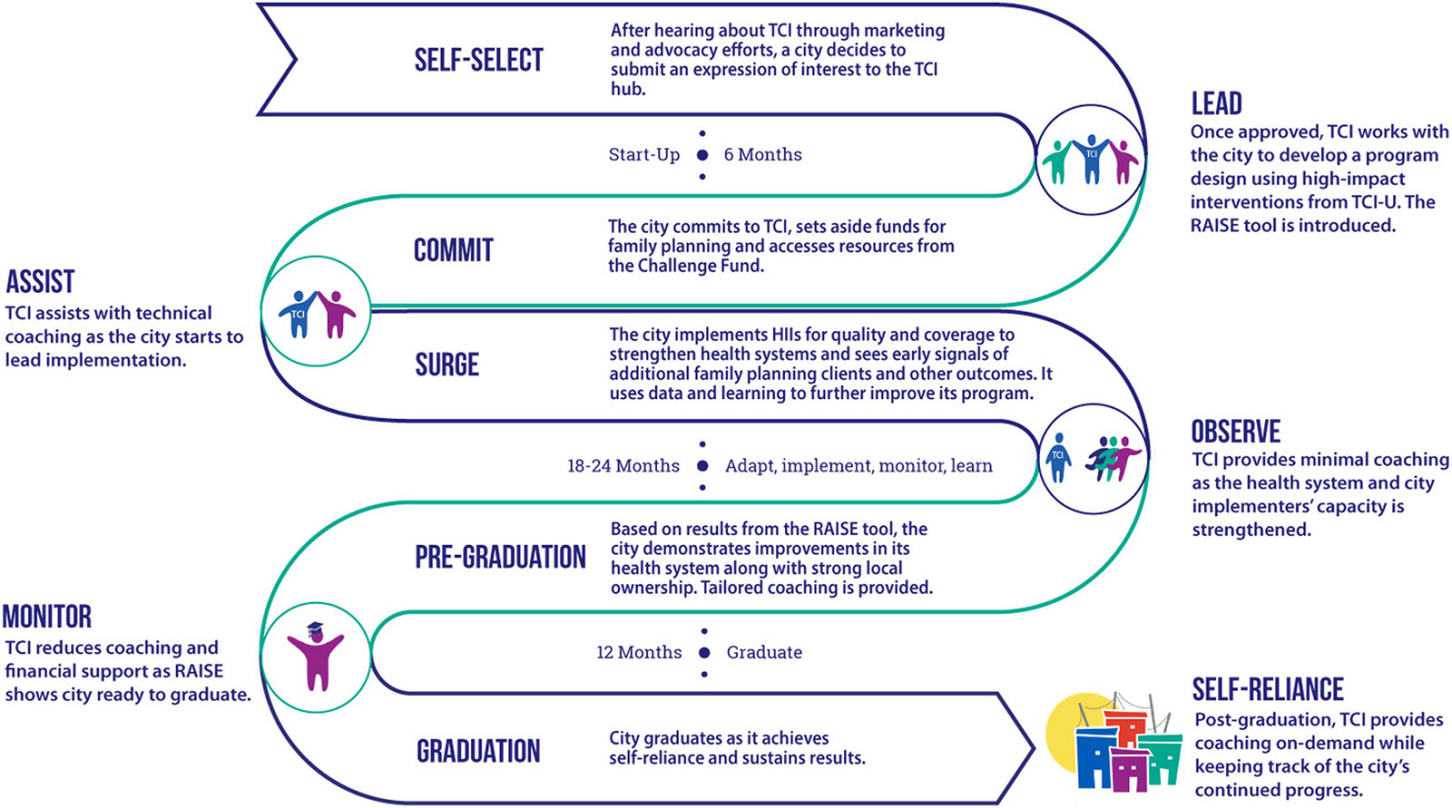
The Challenge Initiative Local Engagement Roadmap Abbreviations: HII, high-impact intervention; RAISE, Reflection and Action to Improve Self-reliance and Effectiveness; TCI, The Challenge Initiative; TCI-U, The Challenge Initiative University.

TCI is a partnership and platform that invited states in Nigeria to apply, pledge annual funding, and commit to assuming a leadership role to receive technical and seed funding support to scale up evidence-based, high-impact FP and adolescent and youth sexual and reproductive health (AYSRH) interventions in a sustainable manner.^15^ A state’s engagement with TCI typically spans 3 to 4 years and goes through 4 broad stages: commit/start-up, surge, pre-graduation, and graduation. Across these stages (Figure 1), TCI’s coaching support, which is initially intense, gradually reduces as systems strengthen and local stakeholders become increasingly capable of independently implementing the FP interventions.

TCI partner states select high-impact practices and other interventions based on their FP needs and adapted to their local health and social context through a robust program design process. The interventions span the 4 thematic areas of advocacy and program coordination; demand generation; service delivery; and monitoring, evaluation, and learning. To implement the interventions, states receive targeted technical and managerial coaching to build capacity, institutionalize the approaches, and strengthen systems to achieve sustainable FP outcomes. States also benefit from joining TCI’s global community of practice, which exchanges lessons learned and shares best practices in delivering FP and health services.

**FIGURE 2:**
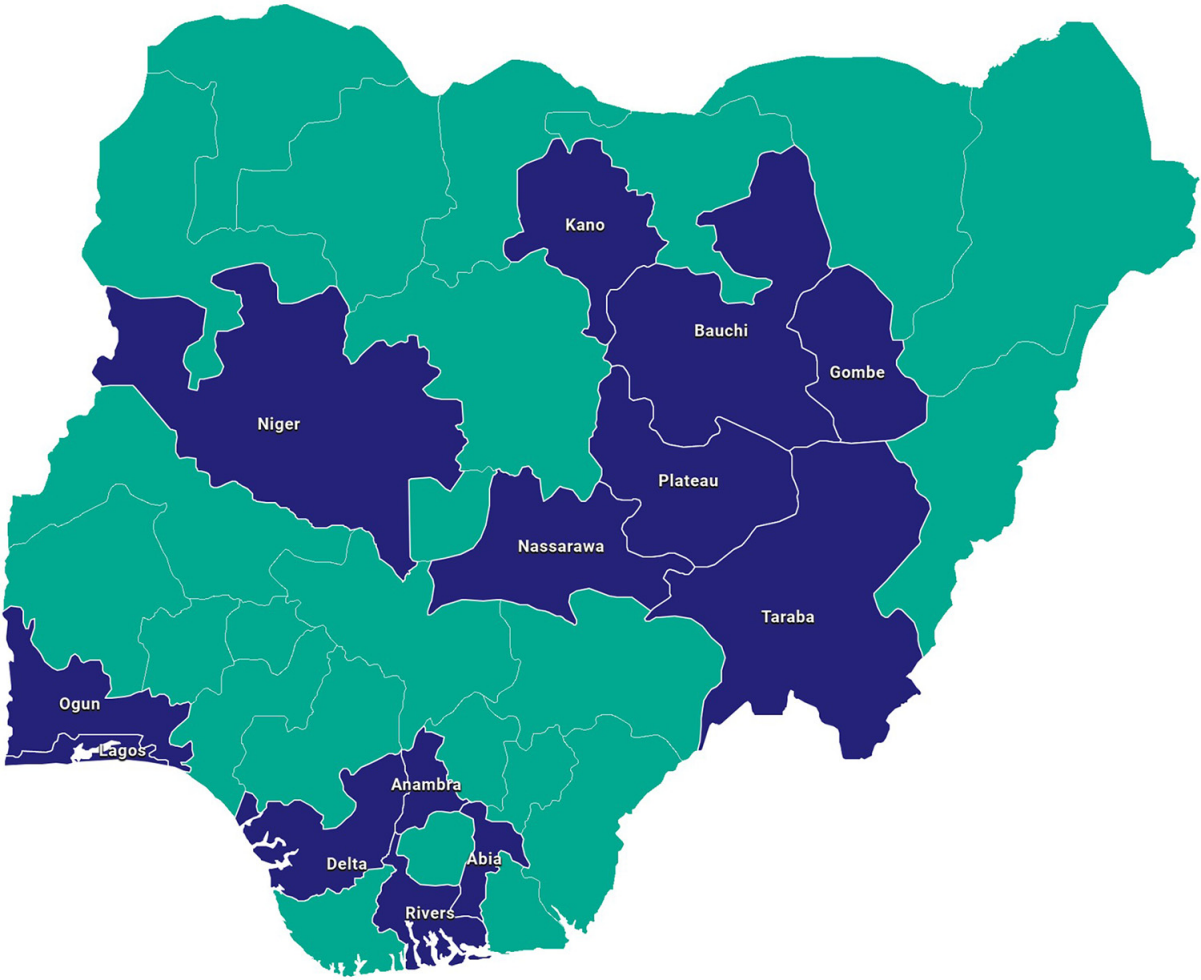
The Challenge Initiative Nigeria Implementation States

Since TCI’s launch in Nigeria in 2017, 13 states have partnered with TCI with engagement initiated over time: 5 states engaged in 2017, an additional 5 in 2018, 2 more in 2020, and 1 in 2021, and 3 states graduated from TCI support in 2021 (Figure 2).

## TCI’S COFINANCING MODEL

TCI supports state governments through a cofinancing model that cultivates program sustainability by incrementally (annually) matching financial contributions from participating state governments. TCI’s cofinancing model addresses several key challenges of health financing in Nigeria: lack of a budget line item, funds allocated but not released, and funds released but not spent for FP. The goal is to institutionalize domestic funding flows in support of FP, improve fiscal responsiveness, and build the capacity of local governments toward more transparent and accountable FP financing.^16^

TCI’s cofinancing model addresses several key challenges of health financing in Nigeria: lack of a budget line item, funds allocated but not released, and funds released but not spent for FP.

TCI’s cofinancing model aims to strengthen state government FP financial commitments by operationalizing cofinancing requirements and tracking the processes leading to states’ achievement of their commitments. The model adapts the concept of “counterpart funding,” which prescribes the financial contributions of partner states based on some measure of predictability for resourcing, scale-up, and systems change. Under the cofinancing model, TCI makes seed funding from its Challenge Fund available to partner states to incentivize the states to commit and release domestic funds for FP. Over the engagement period with TCI, partner state governments are expected to fund an increasing share of the cost of delivering their FP programs while the overall share of the costs borne by the Challenge Fund is gradually reduced. Recurrent government spending on human resources, health systems, delivery of care, and/or general operations does not count toward government domestic funding amounts.

**FIGURE 3:**
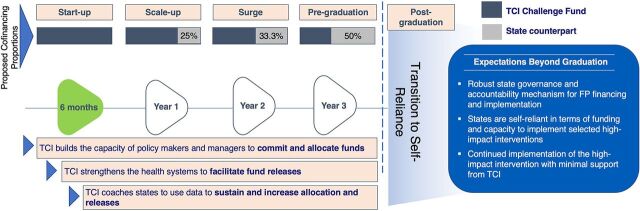
The Challenge Initiative Nigeria Cofinancing Model Abbreviations: FP, family planning; TCI, The Challenge Initiative.

Figure 3 summarizes the cofinancing model’s stages and expectation that the states adopt an increasing share of the implementation costs over the course of their engagement with TCI. The cofinancing model hypothesizes that by distributing the responsibility to finance FP implementation in an incremental manner, governments are more likely to allocate and channel scarce financial resources toward effective FP/RH interventions; external funding can act as a rapid catalyst to address contraceptive needs with equity and quality of care in mind; and FP stakeholders can effectively track FP expenditure trends and use evidence generated by the partnership for sustainable advocacy, planning, and intervention scale-up.

At the beginning of a partnership with TCI, during the program design process, a government proposes how much it will spend for its FP program, which TCI then uses to estimate the Challenge Fund contribution. Subsequently, each year, the state develops an FP workplan, which is embedded in the state’s annual operation plan (AOP). The workplan is then the benchmark with which government-planned counterpart funding is tracked against actual release.

Linked with the high-impact practices and other FP interventions, the cofinancing model serves to strengthen system capacity and institutionalize funding for FP into routine processes, institutions, and funding flows. TCI provides resource mobilization coaching to local health system personnel to ensure the funding required to support the package of interventions is included within a state’s existing FP work plan, AOP, and costed implementation plan. The intervention package becomes the tangible vehicle through which funding is committed and released. Once the funding is included in state-level plans and budgets, FP implementing officers can request funds to implement the interventions such as contraceptive technology updates for managers, competency-based training for service providers, whole-site orientation for FP and AYSRH services, community engagements through targeted demand generation events, in-reaches and outreaches, among others, based on specific state priorities.

The TCI cofinancing model serves to strengthen system capacity and institutionalize funding for FP into routine processes, institutions, and funding flows.

Standardized program tracking and monitoring tools exist within state government systems and are managed and tracked through the Ministry of Budget and Planning. The Ministry is responsible for maintaining aggregate fiscal discipline, allocating resources in accordance with government priorities, and promoting the efficient delivery of services. Government public expenditure reviews are meant to inform strategic planning and budget preparation and to identify ways to improve the efficiency and effectiveness of government resources. However, the data generated through the public expenditure reviews are not available in real time. The TCI cofinancing framework complements the existing framework by providing real-time data on FP expenditure versus budget commitment within a broader and more transparent fiscal space.

Advocacy, led by advocacy core groups (ACGs), plays a significant role in the overall process of mobilizing and institutionalizing funding flows for FP. ACG membership variously draws from government health ministries, religious leaders, reproductive health practitioners, civil society organizations, media, medical associations, female journalists, and others. TCI builds ACG’s capacity to effectively advocate for an enabling policy environment, amplify voices in favor of FP, and mobilize resources for the advancement of FP. This process is designed to foster social accountability through civil society, identify internal and external champions to shepherd the process, and align FP advocacy goals and budgets to existing government policy mandates.

## METHODS

In 2017, TCI Nigeria developed a cofinancing monitoring system that routinely tracks government expenditures, including cash and in-kind contributions, as well as investment of other implementing partners toward implementing FP-specific activities in each state. The monitoring system also tracks Challenge Fund expenditures disaggregated into program-related expenditure (direct activity implementation costs) and technical assistance (human resources, operations, and coaching) costs. This system was synchronized into a web-based dashboard with algorithms coded to estimate cofinancing proportions and project amount of funding that states can leverage in the subsequent year.

First, TCI undertook a 3-year analysis of all budget allocations and releases of partner states from October 2017, when the partnership initially launched with 5 states in Nigeria, to December 2020, by which time 13 states were partnering with TCI. We then analyzed state FP funding contributions in terms of total, proportional, and average contributions budgeted and released per state. Second, the proportion of government counterpart funding based on TCI graduated cofinancing benchmarks across the various implementation stages from start-up to pre-graduation was analyzed. Finally, we analyzed the proportion of state counterpart funding committed (regardless of whether the funds were sourced within or outside the budget line) as well as the extent to which the available TCI funding was used.

On a monthly and quarterly basis, TCI and government counterparts jointly track and review the FP budget line, fund allocations, and expenditures to date. The tracking process helps to check and correct shortfalls from all parties at each stage of the process. Moreover, to determine performance accruals for the next partnership year, TCI periodically tracks and measures progress using 2 additional tracking tools: an implementation progress tracker and a grants performance tracker. The former, which captures expenditures in real time and tracks and visualizes the sum of these transactions over time, provides a format for guiding systematic and actionable monthly reviews of government expenditures based on the set benchmarks and toward the implementation of FP activities as outlined in the state-integrated FP workplan or the AOP. The grant performance tracker assesses and verifies the activities implemented by states each year to determine the amount of TCI funding the state will receive the following year.

## RESULTS

In the last 3 years under review, the following results have been documented based on the use of the cofinancing model.

### Increase in State Family Planning Budget Allocation and Releases

The TCI cofinancing strategy has helped TCI partner states to designate FP budget lines and sustain RH budget commitments and expenditures. During the project period, TCI engaged a total of 13 states in phases, 5 states in phase 1 (2017), another 5 states in phase 2 (2019), and the last 3 states in phase 3 (2020). Of all the 13 states, only 4 states had a dedicated budget line before partnering with TCI. In October 2017, when TCI engaged its first set of 5 states, only 3 states had dedicated budget lines, which increased to 4 states in 2018. By 2020, 10 of 13 partner states had budget lines and released funds for FP. The average amount of funds released per state more than tripled from 2018 to 2019 and decreased moderately in 2020 (Table 1).

**TABLE 1.** Nigerian State Funding Allocation and Release for Family Planning

	2018	2019	2020
Partner states, No.	5	10	13
States with budget lines for FP, No.	4	9	10
States releasing funds, No.	2	8	10
Funds allocated, N (US$)	210,500,000 (584,722.22)	558,309,740 (1,550,860.39)	555,198,495 (1,542,218.04)
Funds released, N (US$)	24,739,158 (68,719.88)	193,672,549 (537,979.30)	169,877,941 (471,883.17)
Allocated funds released, %	12	35	31
Average amount released per state, N (US$)	6,184,789.50 (17,179.97)	21,519,172.11 (59,775.48)	16,987,794.10 (47,188.32)

Abbreviations: FP, family planning; N, Nigerian naira.

The TCI cofinancing strategy has helped TCI partner states to designate FP budget lines and sustain RH budget commitments and expenditures.

Through the cofinancing model, 13 states leveraged a total of N2.03 billion (US$5.6 million) as performance-based co-investment from the TCI Challenge Fund by contributing a total of N1.19 billion (US$3.3 million) of government funding over 4 years (2017–2021). As shown in Table 2, TCI partner states collectively increased their cofinancing commitment as a proportion of overall funding from an average of 32% at start-up to 42% at the pre-graduation phase.

**TABLE 2.** Analysis of TCI’s Challenge Fund Co-Investment Across All 13 Nigerian States Based on Graduated Benchmarks by Stage^7^

Partnership Stage	Challenge Fund, N (US$)	State Released, N (US$)	State Contribution, %	State Contribution Benchmark, %
Start-up^a^	452,817,829 (1,257,827.30)	213,382,013 (592,727.81)	32	0
Scale-up^a^	812,769,147 (2,257,692.08)	432,440,369 (1,201,223.25)	35	25
Surge^b^	568,037,808 (1,577,882.80)	408,008,874 (1,133,357.98)	42	33
Pre-graduation^c^	194,482,944 (540,230.40)	139,398,250 (387,217.36)	42	50
Total	2,028,107,728 (5,633,632.58)	1,193,229,506 (3,314,526.41)		

Abbreviations: N, Nigerian naira; TCI, The Challenge Initiative.

^a^13 states.

^b^10 states.

^c^5 states.

During that period, 65.2% of available Challenge Funds have been accessed, and 78.7% of funds committed by state governments have been released (Table 3). Although all states provided some proportion of counterpart funding, some states provided more funding than others. Of the 13 states, 5 contributed about 60% of the total state co-investment and only 1 state, Plateau, exceeded a 4-year average of 50% (Table 4).

**TABLE 3.** Analysis of TCI’s Challenge Fund Co-Investment With 13 Nigerian States Based on Commitment Versus Releases, by Year

			Partnership Year			
Cofinancing Category	Funding Amount, N (US$)	Proportion Accessed/Released, %	Year 1, %	Year 2, %	Year 3, %	Year 4, %
TCI Challenge Fund available	3,109,227,343 (8,636,743)	65.2	13.1	62.8	65.8	73.7
Total TCI Challenge Fund accessed	2,028,107,728 (5,633,633)					
Government committed fund	1,515,261,026 (4,209,058)	78.7	15.4	71.1	97.7	75.7
Total government cofunding released	1,193,229,506 (3,314,526)					

Abbreviations: N, Nigerian naira; TCI, The Challenge Initiative.

**TABLE 4.** Average Counterpart Contribution by Nigerian States

State	Partnership Duration, Years	Average Contribution, %
Plateau	4	56
Bauchi	4	43
Rivers	4	39
Kano	4	35
Taraba	4	35
Niger	4	32
Delta	4	32
Ogun	4	23
Abia	4	27
Anambra	4	28
Nasarawa	1	52
Gombe	1	14
Lagos	1	75

Regardless of the size of their actual contributions, all states were allowed to leverage Challenge Fund resources as contribution shortfalls were not a basis for terminating the partnership but an advocacy and partnership management mechanism and indicator of government financial commitment.

### Taraba State Case Study

Taraba State in northeastern Nigeria comprises 16 local government areas with an estimated population of 3.2 million people, a modern contraceptive prevalence rate of 10.3%, 13.1% unmet need for FP, and a total fertility rate of 5.3%.^17^

In 2018, local leaders engaged with TCI with the aim of improving and sustaining financing for quality FP services. Before this partnership, the FP fiscal space in Taraba State was characterized by a lack of a dedicated budget line, inconsistent and inadequate release of funds for program implementation, a dearth of advocates for funding within the government sector and communities, and suboptimal fund release for consumables.

Through TCI’s partnership with the states, policymakers, technical officers, and other stakeholders, Taraba State received technical assistance in various capacities that spanned proposal writing, implementing FP interventions, and using data to strategically map and advocate to key government policymakers for the designation of a dedicated FP budget line that engenders fiscal responsiveness and accountability. Taraba State also received TCI support to establish ACGs and budget-tracking teams. These teams, which are a subcommittee of ACGs, track budget commitments and releases and develop scorecards, which show government performance and can be used as an advocacy tool.

**FIGURE 4:**
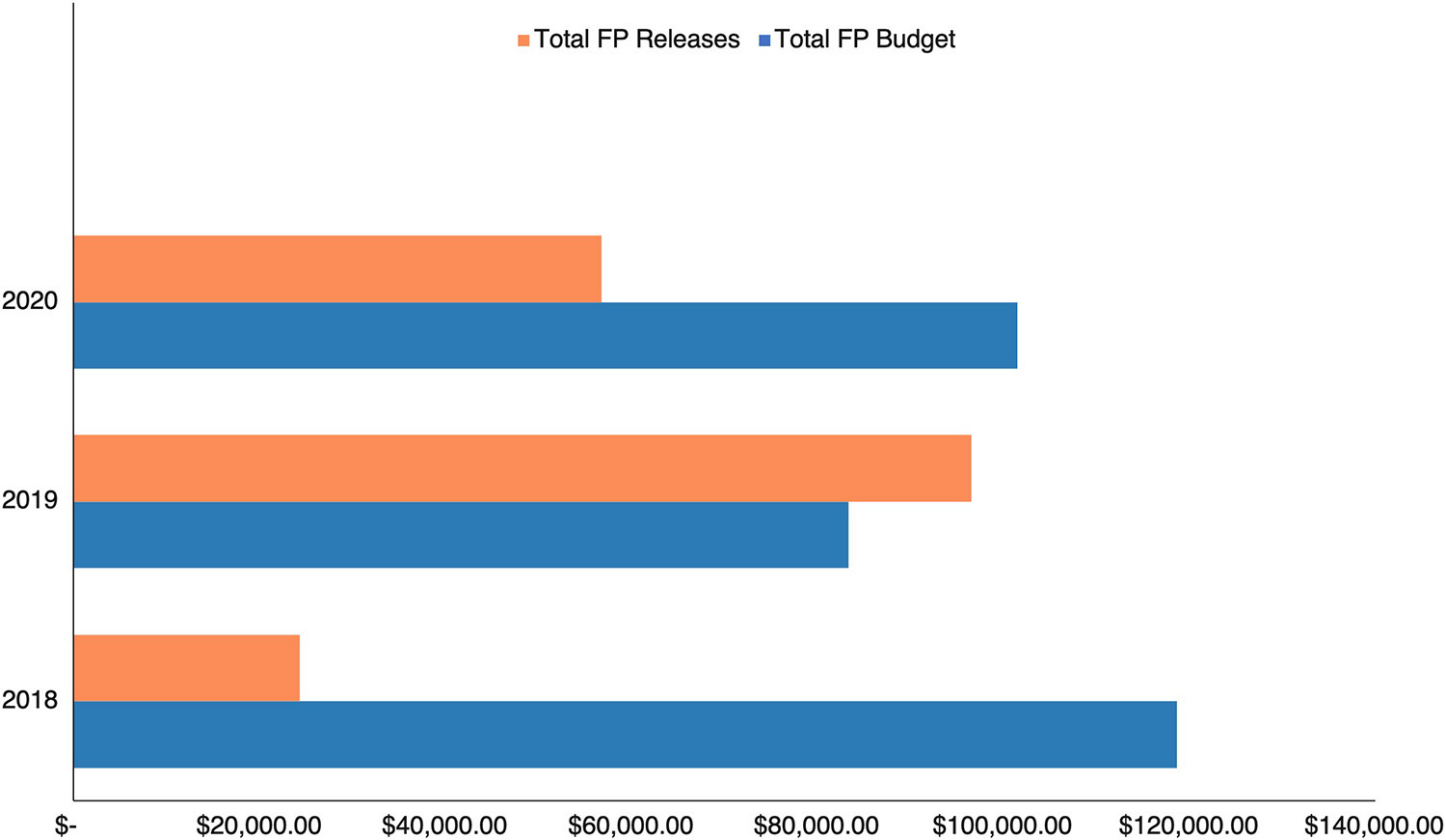
Taraba State FP Budget and Releases Over a Three-Year Horizon Abbreviation: FP, family planning.

After effectively advocating for a dedicated FP budget line, the ACG in Taraba could then successfully advocate to the government to allocate funds through the AOP and release a substantial amount of the funds. This led to a surge in budget allocation and releases in the year following the launch of Taraba’s partnership with TCI in 2018 (Figure 4).

In the 3 years before the TCI partnership, Taraba State lacked a budget for FP. During the first year of the partnership (2018), an FP budget line item of US$118,704.44 was included, though only about 21% of that line item was ultimately released (Figure 4). The following year, while the budget was lowered to US$72,101.35, the actual funds released for FP increased to US$83,549.20—116% of the committed funds. In 2020, the FP budget line item was US$87,856.26, with 56% (US$49,151.54) of the line item released despite increased financial demand due to the COVID-19 pandemic.

The overall increase in funding committed and released in Taraba was catalyzed by the ACG’s persistent advocacy engagement with the government through budget negotiations with key policy decision-makers, budget memo development, strategic meetings, data utilization to support the government’s decision on fund allocation and releases, and increased community demand for contraceptives, especially during the COVID-19 lockdown. Over the 3-year period, client volume at public health facilities more than doubled in Taraba. The ACG presented these data to the state government as evidence for the need for continued and sustained funding for FP, which, in turn, motivated the state government to work toward maintaining the upward trend by sustaining the release of more funds. Most significant was the Taraba State government’s response to the FP commodity stock-out during the COVID-19 lockdown when commodities and consumables were procured to sustain access to quality services by women, men, and young persons who needed contraceptives. Policymakers could now make use of FP service utilization data for planning, budgeting, and informed decisions. The observed trend in budget allocation and releases are sustained in Taraba by the functional ACG, embedded high-impact FP interventions within the AOPs, and regular data and program review meetings to assess progress and course corrections.

The overall increase in funding committed and released in Taraba was catalyzed by the ACG’s persistent advocacy engagement with the government.

## DISCUSSION AND KEY LEARNINGS

There is broad agreement in the literature that government ownership is a prerequisite for the sustainability of FP/RH interventions in developing countries, but less is known about the role and dynamics of government partnerships in driving the process.^17^ Local ownership cannot be meaningful until both government and nongovernmental actors engage transparently and contribute to increased fiscal visibility. The TCI cofinancing model, built on existing government financial processes and institutions, recognizes that only governments can drive program implementation for results while promoting the ownership and sustainability of the program.

The TCI cofinancing model recognizes that only governments can drive program implementation for results while promoting the ownership and sustainability of the program.

The model has proven to be a viable strategy to increase the commitment, release, and spending of FP funds, build relationships among state actors (donors, implementers, and government), and improve accountability through transparent agreements and documentation of commitments and the actions required to fulfill them. Since 2018, increases in FP funding in partner states can be attributed to the resource mobilization coaching process embedded within the TCI cofinancing model; the focus on institutionalization of the innovative practices into existing policies, programs, and processes; and support for ACGs to hold the government accountable on its promises while unlocking additional resources to finance its FP plans. Other states and countries seeking to adopt the cofinancing model should consider the following recommendations from a health systems capacity and operational standpoint.

### Design a Robust Advocacy Strategy

Governments and civil society must deploy policy and program advocacy to ensure states are on a path to ownership and health systems resilience. Because cofinancing does not happen in isolation, TCI works with partner states to design a robust advocacy strategy that includes using appropriate policy frameworks and engagement plans to foster consistent and sustained financing for health. This effort also involves an intentional process of planning and building consensus for the adoption of the model, early relationship building, and collaborations with relevant government and nongovernment partners.

### Institutionalize a Mechanism for Health Financing Transparency, Accountability, and Good Governance

The core of TCI’s work is to build the management and leadership capabilities of states to lead, drive, and own their health programs while supporting their transition to self-reliance and autonomy. In sustaining improvements in the FP financing architecture, governments should be offered technical support in documenting and tracking financial commitments and releases in a way that reflects accountability and fiscal responsiveness and builds a resilient system responsive to the FP needs of its citizens. In addition, state governments and local institutions should be prepared to step up and internalize a culture of anticipating and preparing for donor transition after technical support ends.

The core of TCI’s work is to build the management and leadership capabilities of states to lead, drive, and own their health programs while supporting their transition to self-reliance and autonomy.

### Implement a Coherent Performance Management and Continuous Quality Improvement Mechanism

TCI adopted a comprehensive mechanism for tracking and measuring cofinancing process and outcomes to determine performance accruals for the next partnership year. This mechanism provides a format for guiding systematic and actionable monthly reviews of government expenditures based on set benchmarks and their progress toward the implementation of FP activities as outlined in the state-integrated FP workplan or the AOP. The state commitment is tracked against the matching fund benchmark for the year. The implementation tracker also helps the government track and document other sources of funding, including government development grants, loans, and other intervention funds. The data obtained from these trackers can contribute to building the investment cases for FP, prioritize proven interventions, and demonstrate ownership through state-led implementation built on the TCI model.

## CONCLUSION

As the global funding from donors continues to dwindle, there is increasing need for locally driven financing models that promote ownership and sustainability of FP/RH interventions.^18^ This need has been recently underscored by the 2022 Lancet Nigeria Commission report with its call for governments to lead efforts to improve health financing through approaches such as revenue mobilization and pooling and management of funds.^19^

In this article, we examined TCI’s innovative approach to public financing for FP with a focus on interventions at the subnational levels. Our findings which draw on the learnings of previous health cofinancing mechanisms^11^^–^^13^ suggest that cofinancing strategies, when integrated into existing mechanisms of resource allocation and releases, encourage a higher level of government ownership and sustainability.^20^ Through advocacy and accountability levers, the investment case for FP should be aligned with political incentives within a broader health development financing agenda.^21^^,^^22^

Our findings also highlight the importance of effective tracking of the cofinancing key performance indicators and the need for a robust data management system. As one of the major challenges encountered during the process of design and deployment of the model, TCI Nigeria periodically experienced delays in obtaining critical data for performance analysis. In addition, many states have an ineffective internal audit system that does not allow them to produce timely, functional expenditure reports from core accounting data. This is further compounded by a lack of trust and transparency in documenting actual expenditures. Therefore, routine reporting and data collection systems should be strengthened to improve data visibility for evidence-based planning and decision-making.

The model is one of many factors that lead to positive outcomes. It is as much a tool for transparency and consensus building as it is mobilizing states to achieve benchmark contributions, but it requires strong advocacy and data support to realize gains. The hypothesis on which the cofinancing model was conceptualized focused on 3 main elements—domestic financing, resource optimization and leverage, and fiscal accountability and transparency. The cofinancing model was an ambitious attempt not only to bring more visibility to FP/RH financing in a way that stimulates domestic financing but also to incentivize the government to sustain its investment and programmatic efforts.

However, because of the short period of partnership with the states, we have only 3 years of data for most states. There is great variability in performance among states, and it may be difficult to infer that only this cofinancing mechanism may have been responsible for the results recorded. However, there is still an opportunity to strengthen this mechanism as TCI continues to monitor state financial contributions 12 months post-graduation and will use this information to strengthen the model. The cofinancing strategy introduced here is not a replacement for existing financial planning and program monitoring tools. TCI continues to build government capacity to integrate routine fiscal analyses into existing program planning and monitoring systems, while transferring knowledge to civil society organizations to use the model for budget tracking.

With ongoing review and adaptation of the TCI cofinancing model at the national and subnational levels, it is expected that state governments will be able to commit and release more funds for FP and RH in general by harnessing a wider array of local resources for a more responsive and improved health system. The co-investment leveraged by states can act as a major incentive for domestic financing and budget transparency. This is an area that requires further research and testing.

## Notes

**Peer Reviewed**

**First published online:** December 27, 2022.
